# Vape Shop Employees: Public Health Advocates?

**DOI:** 10.18332/tpc/67800

**Published:** 2017-01-20

**Authors:** Joy L Hart, Kandi L Walker, Clara G Sears, Alexander S Lee, Courteney Smith, Allison Siu, Rachel Keith, S. Lee Ridner

**Affiliations:** 1 University of Louisville, Kentucky, USA

**Keywords:** Public health, electronic cigarette, vape shops, modified e-cigarette, vaping, vape shop employees

## Abstract

**INTRODUCTION:**

E-cigarettes have increased in popularity and given rise to a new type of sales outlet—the vape shop. Expanding on work examining vape shop employee e-cigarette and tobacco attitudes and behaviors^1^, this study examined key messages that vape shop employees communicate to customers.

**METHODS:**

Using informal interviews, observations, and a cross-sectional survey, we examined vape shop employees’ (n=16) perceptions and e-cigarette use. Data were collected in nine vape shops in Louisville, Kentucky. We used open coding to analyze the qualitative interviews, observation notes, and open-ended survey responses. Descriptive statistics were used to analyze survey data.

**RESULTS:**

The findings revealed that nearly all employees were former smokers (93.8%), who now only use e-cigarettes. Over one-third of the employees (37.5%) began using e-cigarettes as a replacement for traditional cigarettes, and 93.8% reported better health (e.g., easier breathing, less coughing) since starting to use e-cigarettes. Although most employees believed e-cigarettes should be regulated, 56.3% thought regulations should be different from those governing traditional cigarettes. Analysis of qualitative data revealed that employees see themselves as health advocates who: **1)** provide instructions on vaping and promote a vape community, **2)** encourage cessation of traditional cigarettes, and **3)** support some regulations.

**CONCLUSIONS:**

The findings reveal that vape shop employees regard e-cigarettes as viable smoking cessation tools and relish their role in assisting others in taking what employees view as positive health actions. Future research addressing communication between vape shop employees and customers, especially related to smoking cessation and health, is needed.

## INTRODUCTION

Since their introduction to the marketplace, electronic cigarettes (e-cigarettes) have rapidly increased in popularity. These battery-powered devices utilize a cartridge or refillable tank housing e-juice (i.e., a liquid mixture) that is heated during use, converting the e-juice into an inhaled aerosol or “vapor”^2^. Several studies document considerable increases in e-cigarette use each year, and some estimates suggest that by 2017, e-cigarette sales could exceed $10 billion^3, 4^. For example, Vapor Corp, one of the leading e-cigarette companies, reported $26 million in 2013 revenues. According to a year-end press release, product placement in over 60,000 stores in the United States and Canada and increases in marketing efforts led to a 56% jump in sales during the final quarter of the year^5^. Further illustrating marketplace popularity, the products have already entered their third generation, moving from the initial cigarette-like (i.e., cigalike) to modifiable devices (i.e., mods), which allow users to customize nicotine and flavors^1, 3^.

Although questions remain about the individual and public health effects of these products as well as their overall safety, growth in sales of e-cigarettes shows that the public is voting acceptance with its wallets. Considerable funding has been devoted to promoting e-cigarettes, thus increasing awareness and use, in several ways. Initially, the products were primarily marketed and sold online, but increased public interest and use led to retail stores stocking the products^6^. Gas stations and convenience stores became frequent purchasing sites. As e-cigarettes continued to evolve and sales soared, new forms of distribution have appeared.

The economic boon associated with the proliferation of e-cigarette use has given rise to a new type of sales outlet, vape shops, which are specialty retailers devoted to the e-cigarette market^1, 6^. Such shops are opening with great frequency; thus, accurate assessments of their market penetration are challenging. Typically, vape shops offer products available online and in traditional stores as well as options to customize the user experience. For example, these independent retailers often sell unique e-liquids that users can select. Additionally, some vape shops encourage customers to participate in a variety of activities, ranging from sampling/tasting opportunities to vaping lounges^6^.

Limited research has examined vape shop employees’ interactions with customers^1, 7^. Although some research has documented that vape shop marketing strategies are similar to tobacco marketing strategies, both practices that are now prohibited (such as outdoor promotion near schools) as well as current ones (such as promotion at bars), little is known about how the employee interface influences customers or how employees describe their role and interactions with potential purchasers^1, 8^. Expanding on research examining vape shop employee e-cigarette and tobacco attitudes and behaviors^1^, this study examined how vape shop employees see their role in serving customers. Specifically, interest centered in understanding the key messages that vape shop employees communicate to customers. This employee-customer interface is especially important as the potential health benefits and dangers of e-cigarettes are not fully understood^9^, vape shop owners have been identified as key sources of information^10^, and vape shop employees communicate with numerous e-cigarette users and potential users. Such communication likely influences selections that customers make, how they perceive e-cigarettes, and how they evaluate the health implications and safety of vaping; thus, the consequences of these interactions are far-reaching.

## METHODS

This research was approved by the university’s Institutional Review Board and conducted in accordance with an approved protocol. Prior to data collection, vape shops across Louisville, Kentucky were contacted and asked to participate in the study. At the time of recruitment, 19 vape shops were identified in the metropolitan area. To increase the likelihood of capturing a sample representative of e-cigarette users in Louisville, we divided the city into geographic sections, and selected shops from each area. We visited five shops per section, but did not recruit participation from all (e.g., low shop traffic). Ultimately, nine vape shops agreed to be part of the study. These shops included the two major local chains (i.e., two shops from one chain and three from the other), with several area stores and considerable local influence and online presence. Since our data collection, the number of vape shops in the city has doubled.

Over a 6-month period in mid-2014, members of our research team made several visits to these stores and recorded observations. Each shop was visited at least three times, typically by a team of two or more researchers, with visits occurring on different days of the week and different times of day. Notes from the observations included information on the level of store traffic, questions asked by shop patrons, answers provided for such questions, advice given by employees, and suggestions made to our research team. In addition to observations, informal, semi-structured interviews with employees (n=12) were conducted and a survey was administered to 16 employees and owners of vape shops. The inclusion criteria for this analysis were that one had to be an employee or manager of the vape shop, at least 18 years of age, and English speaking. All participants were recruited during work hours at the shop.

The vape shop employee survey was investigator generated and consisted of questions on demographics, use behavior (delving into the employee’s traditional cigarette use and e-cigarette use), and perceptions, attitudes, and beliefs (such as health and safety) about e-cigarettes. In addition, an open-ended question at the survey’s end (i.e., What else should we know about e-cigarettes?) allowed the vape shop employees to share additional information with us. Quotes included in this analysis were captured from open-ended items on the questionnaire. Descriptive statistics were calculated using SAS 9.4 (Cary, N.C.), and open coding was used to identify themes in textual data (i.e., interviews and open-ended questionnaire responses). Thematic analysis allows researchers to inductively identify and analyze emergent patterns. The constant comparative method was employed to identify themes and ensure category saturation^11, 12^, and related subcategories were identified using axial coding,^13^ which connects features to a specific idea or context. Three authors coded independently, and when differences in interpretation occurred, the coders discussed these until 100% agreement was reached.

## RESULTS

Participants (n=16) were mostly male (75%), white (93.8%), and young (age ranged from 18 to 44, with a median of 22.7, mean of 28.1, and standard deviation of 9.5). All of the participants had graduated high school with 37.5% of the sample having some college, a 2-year degree, or 4-year degree. The majority of the sample (87.5%) was single (never married or divorced). Vape shop employees also reported their e-cigarette use. They use 30–900 ml a month of e-juice with a median of 180 ml. Most of the employees (75%) used 1–3 mg/ml nicotine in e-liquid and reported fruit (50%) and dessert (50%) as some of their favorite flavors. In addition, employees reported where they most often use e-cigarettes. The top three most popular places to use e-cigarettes were at home (93.8%), at work (86.7%), and in the car (86.7%).

From the observations, interviews, and open-ended responses, the overarching theme was that vape shop employees see themselves as health advocates. This primary theme has three parts. Specifically, vape shop employees believe they are health advocates who 1) Provide instructions on vaping and promote a vape community to keep users safe and improve the vaping experience, 2) Encourage cessation of traditional cigarettes, and 3) Support regulations that protect user safety, encourage tobacco cessation, and limit youth access.

### Provide Instruction and Promote Community

Our observations, interviews, and open-ended survey responses contained numerous references to and explanations of employees’ commitments to educating others, through instructions and fielding questions, and promoting safety and improved vaping. Throughout our study, it was clear that participating vape shop employees see themselves as educational resources for customers. In their views, they provide instructions on product use when purchases are made (including assisting in the selection process and helping customers make good purchase decisions) as well as help with developing and maintaining modified (mod) units (i.e., mechanical devices designed to achieve the type of customization desired by the user). Additionally, the observational data support time spent in conversation and education. For example, across most of our vape shop visits, one or more customers spent at least an hour causally talking with employees, giving employees a chance to share their opinions and suggestions.

All (100%) of our participants use mods. According to their survey responses, most employees (55.6%) learned about mods and how to mod from vape groups and the vape community, with a few learning from YouTube (22.2%) and stores (22.2%). When asked where customers learn about mods, one employee summarized the views of most stating, “The customers learn most about setting them [mods] up in the shops.”

From the perspectives of these employees, there are several reasons to mod. The most frequently mentioned reasons were to customize for myself, as a hobby, and to get a stronger throat hit (i.e., a sensation that originates with the trigeminal nerves, before the chemicals reach the brain, and occurs in throat in the first six seconds after a puff)^14, 15^. Also, mods are regarded as more closely simulating smoking than cheaper e-cigarettes and other cessation devices/methods. According to these employees, another key reason is centered in becoming part of the vape community, which allows interaction, builds connections, and provides pathways to increased information. In one employee’s words, this community is crucial in “mod building and use as a hobby.” Also, important to the employees is that mods allow users to produce bigger clouds, which can be used in vape competitions. Further, many of these employees described hosting vape events, which they see as operating like “a social event” featuring DJs, vape competitions, and food. All of the employees who participated in this study view themselves as members of e-cigarette and mod communities. Examples of community activities include competitions, meet ups, online groups/forums, and email subscriptions. From their vantage point, the community works for the benefit of users, keeping them safe and improving their vape experience.

From the perspective of the employees, e-cigarette users who do not mod are “uneducated vapors,” who are more at risk (e.g., from higher nicotine concentrations, products made by “Big Tobacco”). In general, such users are seen as likely to purchase products (such as cigalikes and pen style) from gas stations and similar outlets, forgoing information and premium product choices available at vape shops. According to vape shop employees, such products are more likely to result in harm to users for several reasons. First, people are not instructed on how to properly use them; thus, they may do so incorrectly. Second, these products are perceived to have higher nicotine concentrations and do not allow users to control nicotine level. Third, there is ambiguity about e-liquid content. For example, one vape employee noted, “the e-liquid contained in such products is manufactured from ingredients of lower quality and may have water substituted for VG [vegetable glycerin] or PG [propylene glycol].” Fourth, these products are controlled by “Big Tobacco,” which is seen as making the products “more dangerous,” “more suspect,” and “more generic.” Further, vape shop employees see such products as resulting in lower user satisfaction.

Nearly all (93.8%) of these employees believe that e-cigarette use has improved their health. In particular, some employees mentioned health benefits from the bigger throat hit from mods. These employees attributed greater lung strength to the “heavier hitting” of vaping (i.e., stronger inhalation needed to produce vapor). One employee also believed that greater dilation in lungs from e-cigarette use helps with allergies.

### Encourage Smoking Cessation

Across the observations, interviews, and questionnaire comments, vape shop employees indicated that they advocated for better health by working to encourage customers to quit smoking traditional cigarettes. They viewed their personal testimonies and stories as a key method to help others stop smoking. Nearly all (93.8%) of the employees were former smokers, and none reported currently using traditional cigarettes. All of the former smokers believed that using e-cigarettes resulted in their cessation success with traditional cigarettes, and over one-third (37.5%) initially turned to e-cigarettes to cut back on or quit traditional cigarettes. In particular, their smoking cessation stories point to the harm in using traditional cigarettes as well as the usefulness of e-cigarettes in smoking cessation. The following quotations are examples of these viewpoints:

“I believe e-cigs saved my life. At the rate I was smoking at 17, I would’ve died before I turned 55.”

“As an ex-pack and a half a day smoker, e-cigs were the only alternative nicotine delivery system that actually satisfied my need, my want to smoke. I’ve used gum, patches, as well as quit cold turkey, and it never worked.”

Because most are former smokers, employees perceive themselves as especially skilled on instructing customers on how to best use e-cigarettes for cessation purposes. Further, for these employees, assisting customers with and advising them on quitting traditional cigarettes equates with promoting health. In short, according to these employees, “They [e-cigarettes] save lives.”

Given their harm reduction views of e-cigarettes (i.e., safer and reinforcing cessation of traditional cigarettes), vape shop employees regularly share perspectives with customers. For example, because, “they [e-cigarettes] do not give off formaldehyde, they are healthier than traditional cigarettes.” Further, some employees asserted that, “People usually think nicotine is bad, but it’s found in fruits and vegetables.” One employee described this information this way:

“PG & VG [propylene glycol & vegetable glycerin], the two main ingredients, have both been deemed safe by the FDA 30+ years ago. PG is the main ingredient in inhalers; it’s also used in hospitals to purify the air and help stop the spread of airborne pathogens. VG is used in fog machines. So if you’ve ever been to a concert where they have fog machines or to a hospital at all, then you have inhaled the basic components of an e-cig.”

Additionally, as noted above, employees share information about mods, which they believe will be especially useful in helping customers to quit traditional cigarettes. In short, in their opinion, the ability to customize assists with success in cessation.

Employees also promote and celebrate customers’ cessation success. For example, they shared information on processes customers used to wean themselves off traditional cigarettes and frequently referenced people they had helped. At some stores, customers are recognized for their successes. For example, one stores features a wall with signatures from customers who quit using traditional cigarettes. According to the employees, this recognition reinforces customers who have stopped using traditional tobacco and motivates others who wish to quit.

### Support Some Regulation

Most of the employees (56.3%) who participated in our study believe that e-cigarettes should be regulated, but not the same way that tobacco is regulated. One summarized the views of many by stating that “They [e-cigarettes] aren’t tobacco or cigarettes;” thus, they should not be treated the same. Although over half support regulation, this view that e-cigarettes and traditional cigarettes are vastly different is the primary reason that more than one-third of employees (37.5%) do not support regulation of any type.

When employees support regulation, they are especially concerned with protecting user safety, encouraging tobacco cessation, and limiting youth access. In terms of user safety, employees who favor regulation want to ensure that e-liquid ingredients are of high quality and that labels convey accurate ingredient information. In particular, they urge caution of e-liquids made in China. One employee summarized the situation this way:

“The biggest controversy over e-cigs comes down to the liquid. Most e-liquid manufacturers in the USA regulate themselves, by using ISO certified labs and carefully monitoring their ingredients. Although this is the case for most, some USA stores outsource their liquid from China. This is where the e-cigs have 10 times more cancer causing ingredients than traditional cigs. Because China does not regulate themselves, they use cheaper, unsafe ingredients. This is why I believe there does need to be some regulation, just not the regulations lawmakers have in mind. E-cigs have also taken a huge amount of business away from tobacco companies who also make the Vuse, Blu, MarkTen.”

These employees were also skeptical of “Big Tobacco” and its role in the e-cigarette industry. This skepticism arises from several concerns. First, these vape shop employees are cautious about e-cigarettes produced by tobacco companies. Several employees expressed distrust of such products. Further, they do not believe that tobacco companies promote smoking cessation or have this outcome as a goal. Additionally, there is considerable concern that large tobacco companies are working to push smaller vape shops out of the market. For example, one employee suggested that, “Several states, like Indiana, have tried to pass unconstitutional laws pretty much banning all e-cigs except for the devices created by tobacco companies.”

These employees also expressed concern about overall safety and limiting youth access. For example, employees support childproof lids, and the shops voluntarily used these lids on any house-made e-juices. Every shop displayed signs indicating no sales to individuals under 18 years of age, and our research team witnessed employees carding customers to determine age.

## DISCUSSION

Based on the analysis of the observations, interviews, and open-ended questionnaire responses, vape shop employees saw a key component of their work as advocating for customer health and safety. The three themes that emerged in our analysis underscore the employees’ viewpoints on and commitment to working for public health.

The employees participating in this study perceive e-cigarettes positively. They demonstrate positive perceptions through frequent consumption; in fact, most employees vaped throughout the day while working. Further, their personal experience and use provide them a base of information to draw from in educating customers. For customers, the employees serve as educational resources, which parallels findings in a recent study of vape shop owners^16^. Defining customers who make purchases in gas stations as “uneducated” and critical of Big Tobacco’s strategies in the e-cigarette market, these vape shop employees are ready to assist customers with the transition to mods. They are happy to do so because they believe moding improves health and assists with smoking cessation as well as provides a superior experience and keeps Big Tobacco from controlling the market.

Because many are former smokers, vape shop employees’ personal testimonies and stories may be powerful means to encourage smokers of traditional cigarettes to try to quit and to believe that they can be successful through the use of e-cigarettes. In the view of these employees, smoking traditional cigarettes is unhealthy, but vaping saves lives. Further, through effective moding, traditional cigarette users can increase their chances of successful cessation, and these employees are happy to assist with information and suggestions.

These employees are members of e-cigarette and moding communities, and it was clear that they serve as gatekeepers in these communities. For example, once trust was established and our data collection was approved, they actively participated. The employees also made suggestions to our research team on how to access the community and additional areas of study. When observing store interactions, it was clear that customers paid careful attention to what employees told them and that many wanted to learn additional information.

All of our interactions with these employees suggest that they are acting in good faith, assisting customers to the best of their abilities and according to their opinions and beliefs. Based on their experiences with and judgments of e-cigarettes and traditional cigarettes, they believe that they advance individual and public health when they help customers with mods, and they enjoy doing so. Their personal stories of smoking cessation, improved health, and strong community are told with conviction and seem believable and thus likely persuasive.

The safety and health of e-cigarette users appears to be a common area of concern for both vape shop employees and FDA regulators. Providing vape shop employees and owners educational resources on FDA regulations^16^ that detail how these rules seek to protect customers may help employees better understand areas of common interest (i.e., of regulators, vape shops, and e-cigarette users) as well as increase the likelihood that employees will support and comply with new regulations.

A considerable body of work in health communication examines the role that narrative can play in processing and accepting health-related information^17–19^. Narrative can both reduce message resistance as well as boost persuasion^20^. Further, in the case of vape shop employees, narratives, delivered as personal testimonies to customers, likely increase believability and persuasiveness. Because these employees strive to help customers make good purchasing decisions, based on what the employees view as good, it seems likely that their personal testimonies, opinions of safety measures, and stories could influence customer views^1^. Shaping narratives to explain the reasoning behind FDA regulations and highlight the importance for customer safety may decrease some resistance to new regulations by the vape shop employees.

Further, because vape shop employees believe that e-cigarettes are safer than traditional cigarettes, overestimating cessation and health benefits and underestimating potential dangers of e-cigarettes are possible^1^, especially when the body of research on overall outcomes is still being amassed. Although these employees may intend to achieve public good, the outcomes of their recommendations are not yet known. Therefore, communicating scientific findings to public audiences, such as vape shop employees and groups most likely to use e-cigarettes, is important in ensuring that facts are appropriately conveyed and the potential for misunderstanding is reduced^16^. Such campaigns should involve traditional communication channels as well as newer social media ones^16^.

Although this study sheds light on vape shop employee-customer interactions, several limitations are present. First, our sample size is small and the data were collected in one metropolitan area. Thus, the findings may not be broadly generalizable. Despite the relatively small employee sample size, however, the effects of these communications are substantial, as each employee talks with a number of customers each week and vape shop employees are key sources of e-cigarette information. Second, the self-reported data from employees may be subject to recall bias. To lessen the potential for such effects, we asked about recent experiences and use.

Future research should examine customer perceptions of vape shop employee communication. This exploratory study examined how employees perceive their role and interactions with customers. Additional inquiry could evaluate how customers assess factors such as believability and utility in employee messages. Further, effective health communication campaigns are needed to raise public awareness, including that of vape shop employees, on the research findings on vaping (both what is known and the degree of scientific uncertainty). With such understanding, employees can better assist customers and customers can make more informed decisions.
